# Acute exercise as a modifier of neocortical plasticity and aperiodic activity in the visual cortex

**DOI:** 10.1038/s41598-023-34749-w

**Published:** 2023-05-09

**Authors:** Claire J. Cadwallader, Jennifer Steiniger, Patrick S. Cooper, Shou-Han Zhou, Joshua Hendrikse, Rachael L. Sumner, Ian J. Kirk, Trevor T.-J. Chong, James P. Coxon

**Affiliations:** 1 School of Psychological Sciences, The Turner Institute for Brain and Mental Health, Monash University, Victoria, 3800 Australia; 2 School of Psychology, James Cook University, Townsville, QLD 4810 Australia; 3 School of Pharmacy, The University of Auckland, Auckland, New Zealand; 4 School of Psychology, The University of Auckland, Auckland, New Zealand; 5 Department of Neurology, Alfred Health, Melbourne, VIC 3004 Australia; 6 Department of Clinical Neurosciences, St Vincent’s Hospital, Melbourne, VIC 3065 Australia

**Keywords:** Neuroscience, Physiology

## Abstract

Long-term potentiation (LTP) is a form of neuroplasticity commonly implicated in mechanistic models of learning and memory. Acute exercise can boost LTP in the motor cortex, and is associated with a shift in excitation/inhibition (E:I) balance, but whether this extends to other regions such as the visual cortex is unknown. We investigated the effect of a preceding bout of exercise on LTP induction and the E:I balance in the visual cortex using electroencephalography (EEG). Young adults (N = 20, mean age = 24.20) engaged in 20 min of high-intensity interval training (HIIT) exercise and rest across two counterbalanced sessions. LTP was induced using a high frequency presentation of a visual stimulus; a “visual tetanus”. Established EEG markers of visual LTP, the N1b and P2 component of the visual evoked potential, and an EEG-derived measure of the E:I balance, the aperiodic exponent, were measured before and after the visual tetanus. As expected, there was a potentiation of the N1b following the visual tetanus, with specificity to the tetanised stimulus, and a non-specific potentiation of the P2. These effects were not sensitive to a preceding bout of exercise. However, the E:I balance showed a late shift towards inhibition following the visual tetanus. A preceding bout of exercise resulted in specificity of this E:I balance shift to the tetanised stimulus, that was not seen following rest. This novel finding suggests a possible exercise-induced tuning of the visual cortex to stimulus details following LTP induction.

## Introduction

Cardiorespiratory exercise is a modifiable lifestyle factor with the capacity to influence neural processes underlying cognition. One line of investigation suggests that exercise can augment long-term potentiation (LTP), a form of neuroplasticity thought to be important for the encoding and storage of information in the brain^1,2^. LTP is a long-lasting increase in the strength of synaptic communication between neurons, induced experimentally by repeated, high frequency activation of an afferent neural pathway^1^. Although LTP is commonly associated with hippocampal function and memory in animal models, it is a crucial brain-wide process^3–5^. Exercise is known to have diffuse effects throughout the brain that are likely to support the LTP process, such as increased concentrations of brain-derived neurotrophic factor (BDNF)^6^, glutamate and gamma-aminobutyric acid^7^, and an increase in cerebral lactate uptake^8^. Thus, exercise is thought to create an environment that is conducive for LTP.

Advances over the last two decades have seen the emergence of non-invasive paradigms that allow the indirect measurement of LTP in humans (as reviewed by Refs.^9,10^). Using these methods, it has been demonstrated that an acute bout of high-intensity interval training (HIIT) exercise can augment LTP-like potentiation in an unexercised area of the motor cortex^11,12^. Further investigation into the mechanisms that might underlie this exercise-related augmentation of LTP using transcranial magnetic stimulation (TMS) found that there was decrease in short intracortical inhibition and an increase in intracortical facilitation^13–16^, indicating a shift in the cortical excitation-inhibition (E:I) balance towards excitation^13^. Although these results demonstrate the potential benefits of an acute bout of exercise for LTP and the mechanisms that may underlie this process, the effects of acute exercise on LTP induction beyond the motor cortex are unknown in humans.

In an electroencephalography (EEG) paradigm first developed by Teyler and colleagues^17^ the falling edge of the N1 component of the visual evoked potential (VEP), the ‘N1b’, exhibits an LTP-like potentiation (i.e. an increase in N1b amplitude). The increase occurs following a visual tetanus; a high frequency (~ 9 Hz) presentation of a visual stimulus, and is observed bilaterally over the occipito-parietal cortex, centred approximately around electrode P7 and P8 (for review see Ref.^9^). Importantly, this potentiation has demonstrated key features of Hebbian LTP, including input specificity^18–20^ and N-methyl-D-aspartate (NMDA) dependence^21^. Subsequent visual LTP studies have also shown a long-lasting potentiation of the P2 amplitude in the central occipital region (electrode Oz/POz)^20,22^. It is currently unknown whether acute exercise can impact the magnitude of LTP expressed by these EEG markers in the visual cortex.

Furthermore, a novel approach to measuring the E:I balance with EEG recordings, the 1/f aperiodic exponent, was introduced recently^23^. Aperiodic activity is characterised as EEG signal without a characteristic oscillatory frequency^23^. This activity displays a 1/f-like distribution, where power across the increasing frequency band decreases exponentially. When modelled, the aperiodic offset (the intercept) summarises overall broadband power across the power spectrum, and has been proposed to reflect total neuronal population spiking^24,25^. The aperiodic exponent (the slope), is a marker of cortical excitation inhibition (E:I) balance^23^. A steeper slope (larger exponent) indicates E < I, whereas a flatter slope indicates E > I. Current evidence shows that the aperiodic exponent is modulated by age (less inhibition in older adults)^26^, and by anaesthetic drugs acting on GABA receptors (propofol)^27^. The effects of exercise, or a visual tetanus, on the 1/f aperiodic exponent is entirely unknown.

In this EEG study we aimed to use the visual LTP paradigm to investigate the effect of an acute bout of HIIT exercise on the magnitude of LTP expressed in the visual cortex. We hypothesised that there would be an increased magnitude of LTP following a preceding bout of HIIT exercise, compared to an equivalent period of rest. Furthermore, given that LTP is known to alter the E:I balance^23^, our second aim was to investigate the effect of HIIT exercise and the visual tetanus on cortical E:I balance, as indexed by 1/f aperiodic EEG activity. We expected that this exercise-related boost in LTP would be associated with a shift in the E:I balance towards excitation.

## Method

### Participants

Twenty healthy young adults (12 females, 1 left-handed, age range = 18–31 years old, M = 24.20, SD = 3.56) were recruited through advertisement at Monash University in Melbourne, Australia. Participants had no known history of neurological or psychiatric conditions, and had normal or corrected to normal vision. Participants were also screened for contraindications to exercise (e.g. cardiovascular conditions, musculoskeletal injuries) with the compulsory stage one of the Sports Medicine Australia Adult Pre-Exercise Screening System (2011)^28^. Participants had an average body mass index of 22.94 (SD = 2.96) and average resting heart rate of 71.85 beats per minute (SD = 7.22). All participants were asked to refrain from vigorous physical activity in the 48 h prior to their sessions. Written informed consent was provided by all participants. This study was approved by the Monash University Human Research Ethics Committee (Project ID 18530) and was conducted in line with the requirements of the National Statement on Ethical Conduct in Human Research.

### Apparatus

Both the HIIT exercise and rest condition in this study were conducted on a Wattbike stationary exercise bike (Wattbike, Geelong, Australia). Heart rate was measured using a Polar H10 chest strap heart rate monitor, and was tracked using the Polar Beat mobile app (Polar Electro, Finland).

A 64-channel Ag/AgCl electrode cap (ActiCHamp Plus, Brain Products, Germany) with a 10–20 system montage was used to record continuous EEG data at a 500 Hz sampling rate (low cut-off: DC; high cut-off: 140 Hz), using an ActiCHamp amplifier and BrainVision Recorder software (Brain Products, Germany). Electrode impedances were kept below 10kΩ^29^. All EEG recordings were conducted with an online reference at FCz, and later re-referenced to the average of all channels during pre-processing.

### Experimental procedure

**Figure 1:**
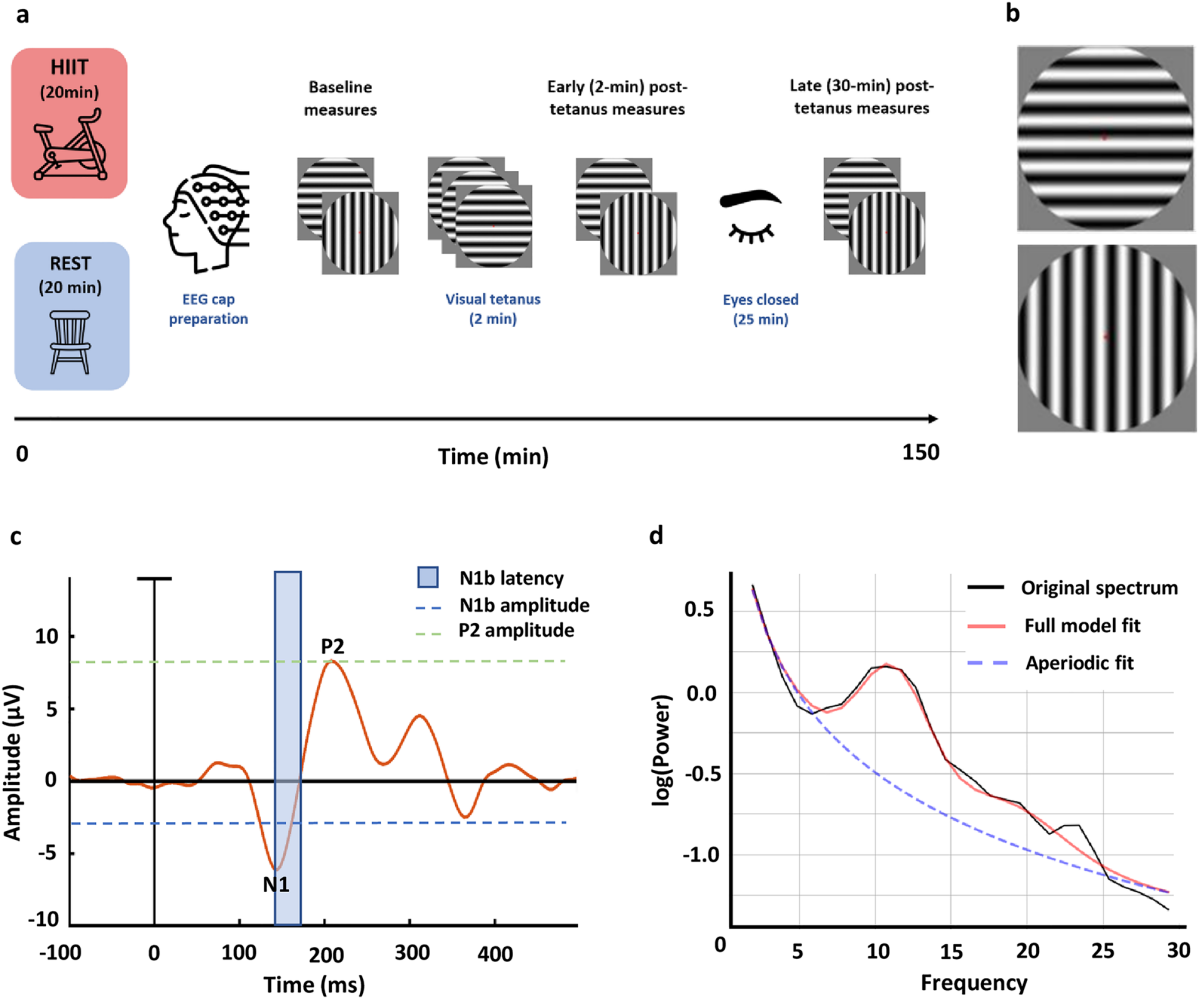
(**a**) Schematic of the experimental procedure. (**b**) The visual stimuli used in the paradigm consisted of a vertical and horizontal circular sine grating. Only one of these stimuli were presented during the visual tetanus, while the other served as the control stimulus. (**c**) An example VEP taken from an individual participant in the baseline measurement block, HIIT condition. The N1b was measured as the mean amplitude of the section of the VEP extending from the peak of the N1, to the midpoint between the N1 and the P2 peaks. The peak amplitude of the P2 was also recorded. (**d)** An example of the FOOOF model fit for an individual participant in the baseline measurement block.

The experimental procedure is shown in Fig. 1a. Each participant completed two sessions separated by at least 72 h. In one session participants completed a 20-min bout of HIIT exercise. In the other session, an equivalent bout of rest was performed. Exercise protocols are described in more detail below. The order of these conditions was counterbalanced across participants.

#### HIIT exercise protocol

Twenty minutes of HIIT exercise was performed by participants on a stationary exercise bike^13,16,30^. The participant’s heart rate was measured throughout (Polar H10, Polar Electro, Finland). Exercise intensity was individualised for each participant based on target percentages of their heart rate reserve (HRR). HRR was defined as the difference between the participant’s resting heart rate (RHR, obtained while sitting), and age-predicted maximum heart rate (HR_*max*_), where HRmax was calculated as *HR*_*max*_ = *208 − (0.7* × *age)*^31^.

Prior to the beginning of the HIIT exercise, the participant engaged in a brief three-minute warm up at an easy pace of their choosing. Following the warm-up, participants cycled in alternating epochs of moderate intensity (3 min each) and high intensity (2 min each) for a total of 20 min. Performance data is shown in Table 1. Participants were instructed to cycle at a comfortable cadence and to focus on maintaining this throughout the 20-min. Exercise intensity was modified by the experimenters via adjustment of the resistance on the bike, and by asking the participant to increase or decrease their cadence slightly, with reference to the heart rate targets. Target heart rate for the moderate intensity blocks was 50–55% of HRR. Target heart rate values for the four high-intensity blocks were 75%, 80%, 85% and 90% of HRR, respectively. Heart rate, workload in watts and a subjective rating of physical exertion using the Borg Scale (range 6–20)^32^ were recorded at the end of each minute. Upon completion of the exercise, the participant was cooled down in front of a fan for 10 min to reduce sweating. A hair dryer on a cold setting was used to dry the hair in preparation for EEG recording.

**Table 1:** HIIT exercise performance.

Time (min)	Mean %HRR	Mean RPE	Mean power:weight (watts:kg)
0–3	44.98 (10.93)	8.79 (2.15)	0.88 (0.23)
4–5	63.01 (12.77)	11.43 (3.57)	1.60 (0.45)
6–8	58.22 (16.88)	10.46 (2.17)	0.91 (0.19)
9–10	80.40 (13.23)	13.92 (1.97)	2.02 (1.01)
11–13	64.73 (17.01)	11.51 (1.66)	0.87 (0.20)
14–15	86.17 (9.26)	15.23 (2.25)	2.17 (0.94)
16–18	65.30 (15.17)	12.43 (1.91)	0.86 (0.20)
19–20	95.54 (9.97)	17.38 (1.66)	2.73 (0.71)

%*HRR* percentage heart rate reserve, *RPE* rating of perceived exertion (6–20).

Standard deviation is reported in brackets. HIIT exercise involved alternating blocks of 3 min of moderate intensity cycling and 2 min of high intensity cycling, for a total of 20 min.

Data recorded for high intensity blocks was taken from the end of the final minute of each block.

#### Rest protocol

Participants sat on the stationary exercise bike and were instructed to turn the pedals over at a very low cadence for 20 min. The goal of this session was to keep the participant’s heart rate within the range of 10 beats per minute from their RHR (mean RHR = 71.85 bpm, SD = 7.22; mean heart rate during the rest protocol = 85.29 bpm, SD = 7.16). This control condition was designed to account for posture and lower limb movement across the two sessions.

#### Visual LTP paradigm

Data recordings commenced within 30 min of exercise or rest completion. The paradigm involved viewing blocks of stimuli while EEG data was recorded. The visual stimuli were two black and white circular sine gratings (9.6 cm diameter) with a spatial frequency of one cycle per degree of visual angle (Fig. 1b). These stimuli differed only by grating orientation; one with horizontal and one with vertical gratings. Stimuli were presented at a viewing distance of 57 cm (subtending 9.6 degrees of visual angle), in the centre of a Dell 27-inch digital computer monitor screen (2560 × 1440-pixel resolution, 144 Hz refresh rate), against a grey background. Throughout stimulus presentation, a red fixation dot was present at the centre of the screen and participants were instructed to remain fixated on this. Stimulus presentation was controlled using the Psychophysics Toolbox (Brainard and Spatial Vision, 1997) in MATLAB (MathWorks, Inc).

To establish baseline visual evoked potentials, participants viewed two blocks of 240 stimulus presentations, each block comprising 120 presentations per stimulus orientation in a randomised order. Stimuli were presented for 33 ms each, at a jittered inter-stimulus interval of 0.67–1 Hz with each block lasting approximately 4 min. This was followed by a ‘visual tetanus’ consisting of 1000 presentations of a single visual stimulus i.e., *either* the vertical or horizontal visual stimulus. The stimuli in the visual tetanus were presented for 33 ms each, at a jittered inter-stimulus interval of approximately 8.6 Hz^18^. Following the visual tetanus, a 2-min break (eyes open) was given to allow retinal after-images to dissipate. Next, the early post-tetanus timepoint was acquired comprising two more blocks of stimulus presentations, as per baseline. Participants were subsequently instructed to close their eyes and sit quietly for 25 min. Finally, the late-tetanus timepoint was acquired, comprising another two blocks of stimulus presentations.

### Analysis

EEG data was processed using EEGLab (version 14.1.1) and the ERPLab plugin toolbox (version 7.0.0) in MATLAB (version R2017a). The time-series for electrodes with significant noise were replaced by interpolation from neighbouring electrodes using spherical spline interpolation. The data was high pass filtered (0.1 Hz), re-referenced to the average of all electrodes, and segmented into 600 ms epochs comprising 100 ms prior to stimulus onset and 500 ms after stimulus onset. Baseline correction was applied using the 100 ms pre-stimulus period. Artifacts were detected and removed using FastICA and auto component selection. Removal of components was verified by visual inspection. The remaining epochs were visually inspected for residual noise and deleted where appropriate. Data for each participant was averaged according to Session (HIIT or rest), Time (baseline, early post-tetanus or late post-tetanus) and Stimulus (tetanised or non-tetanised).

#### Analysis of visual evoked potentials

The analysis of VEPs was conducted using a hypothesis-driven approach based on previously established VEP markers that show LTP-related change using this paradigm; the N1b and the P2 component, and established methods of obtaining these markers (for review see Ref.^9^) (Fig. 1c). As has been previously defined (e.g. Refs.^18–20^), the N1b was measured from bilateral parieto-occipital (LO) regions; electrode chosen for each participant individually (search space, left: P5, P7, PO7; right: P6, P8, PO8). The N1b was defined as the mean amplitude of the section of the VEP extending from the peak of the N1, to the midpoint between the peak of the N1 and the peak of the P2 (average N1b latency: 144–175 ms). To select the electrodes in an unbiased manner, each participant’s data was averaged across HIIT and rest sessions, and a difference wave between early post-tetanus and baseline was created. Electrodes were selected based on visual inspection for the most prominent negativity in the difference wave in the left and right lateral occipital region, and consistent with previous studies, these centred around approximately electrode P7 and P8 in our sample^9^. N1b measures were then taken for each VEP in the HIIT and rest sessions separately, using the participant’s average N1b latency window for each electrode. The P2 component was measured from the central occipital electrode showing the largest P2 amplitudes across participants, POz^22^. The P2 peak was identified for each VEP, for each participant, and the peak amplitude was recorded.

#### Analysis of aperiodic activity

To characterise aperiodic activity in our participants, neural power spectra were calculated for each EEG epoch by means of Fourier Transforms using Welch’s Method (‘pwelch’ function in MATLAB). The neural power spectra were then averaged for each participant according to exercise condition, stimulus and time point. The “Fitting Oscillations & One Over f” (FOOOF) toolbox (version 1.0.0) was subsequently used to parameterize the neural power spectra. The FOOOF toolbox models the full power spectra as a combination of periodic activity (i.e., neuronal oscillations) and aperiodic activity (i.e., non-frequency specific activity) (Fig. 1d). When the periodic component is removed from the model, this allows for estimation of aperiodic components with a high degree of independence from oscillatory activity^23^. The aperiodic activity (L) is modelled by FOOOF according to:

$$L\left( F \right) = b - \log \left( {F\chi } \right)$$

where b is the broadband ‘offset’, χ is the ‘exponent’ of the aperiodic fit, F is the array of frequency values^23^. Settings for the algorithm were: peak width limits (0.5, 12.0); max number of peaks (inf); minimum peak height (0.0); peak threshold (2.0); and aperiodic mode (‘fixed’). The FOOOF model parameterised the power spectra across a frequency range of 1.953–34.180 Hz. For each participant the electrodes of interest corresponded to the same electrodes in the analysis of VEPs described above.

## Results

### N1b

**Figure 2:**
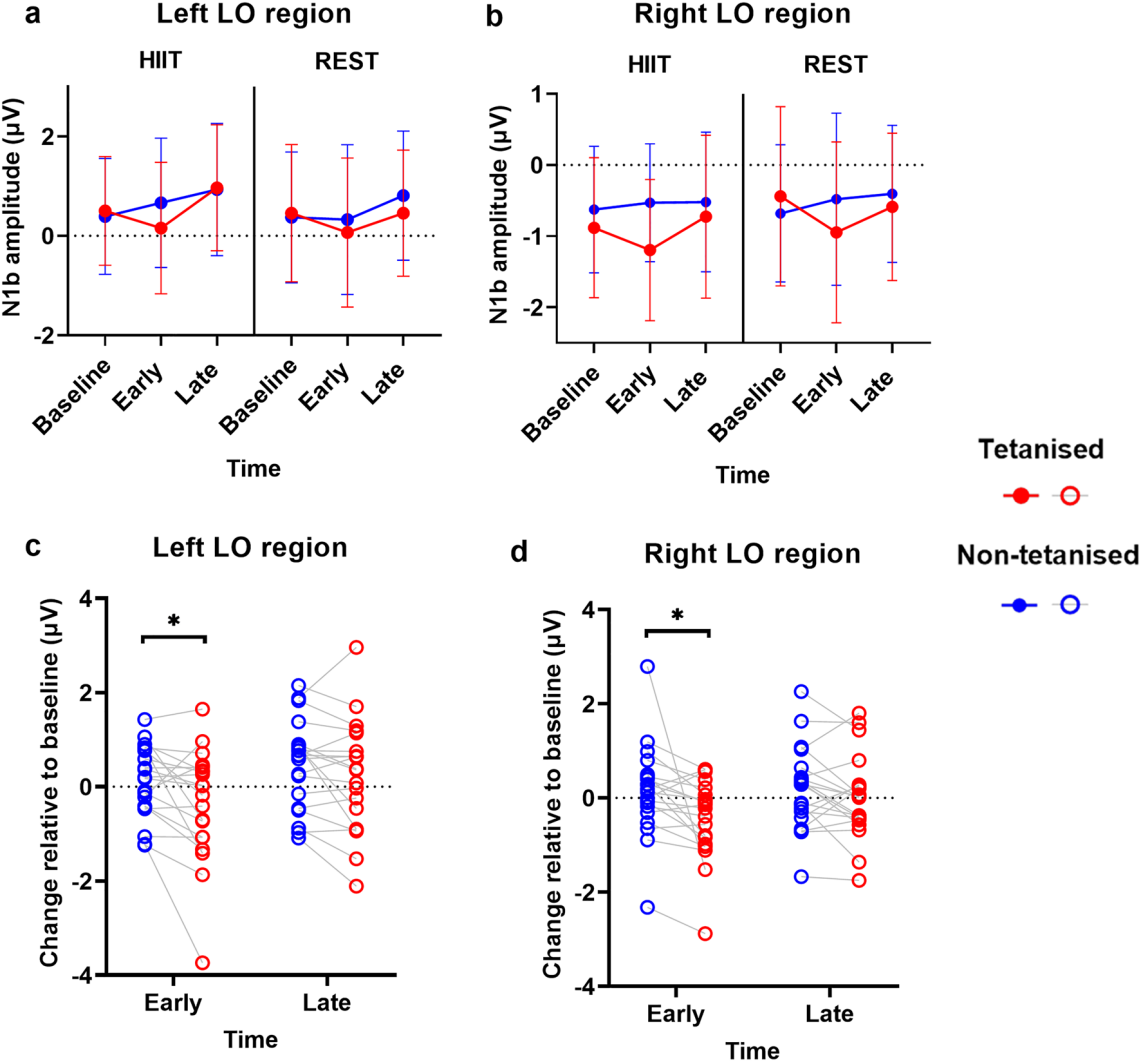
(**a**) N1b amplitude in the left LO region. (**b**) N1b amplitude in the right LO region. (**c**) and (**d**) N1b amplitude change relative to baseline. Individual data points are plotted. Data is collapsed across levels of Session. *p < .05; error bars show 95% confidence interval.

The ANOVA for the N1b amplitude in the left LO region (Fig. 2a) revealed a two-way interaction between Stimulus (tetanised and non-tetanised) and Time (baseline, early post-tetanus and late post-tetanus), F(2,38) = 3.742, p = 0.033, η^2^p = 0.165. Similarly, the ANOVA for the N1b amplitude in the right LO region (Fig. 2b) also showed a two-way interaction between Stimulus and Time, F(2,38) = 4.726, p = 0.015, η^2^p = 0.199. No other effects were significant (all p > 0.056).

To explore these interactions further, data were collapsed across the HIIT and rest sessions. N1b data at baseline was then subtracted from the early post-tetanus and late post-tetanus time points, and two paired t-tests were conducted to assess N1b potentiation relative to baseline for the tetanised stimulus compared to the non-tetanised stimulus (Fig. 2c,d). Results of all post-hoc paired t-tests are shown in Table 2. As expected, in both the left and right LO regions, the potentiation of the N1b amplitude at the early post-tetanus time point (relative to baseline) was greater for the tetanised stimulus, compared to the non-tetanised stimulus (p = 0.026) There were no significant differences at the late post-tetanus time point.

**Table 2:** Post hoc paired t-tests.

	ROI	Comparison	Mean	SD	p	d
N1b	Left LO	Δ EarlyTet – Δ EarlyNontet	− 0.481	0.887	0.026*	0.54
		Δ LateTet – Δ LateNontet	− 0.256	0.640	0.090	0.40
	Right LO	Δ EarlyTet – Δ EarlyNontet	− 0.561	1.005	0.022*	0.56
		Δ LateTet – Δ LateNontet	− 0.188	0.618	0.190	0.30
P2	Central Occipital	Baseline – Early	0.157	1.342	> 0.999	0.03
		Baseline – Late	− 0.901	1.342	0.014*	0.17
		Early – Late	− 1.058	1.342	0.003*	0.20
AE	Central Occipital	For Rest: Δ Early – Δ Late	− 0.096	0.094	< 0.001**	0.76
		For HIIT: Δ Early Nontet – Δ Late Nontet	− 0.071	0.109	0.032*	0.66
		For HIIT: Δ Early Tet – Δ Early Nontet	0.003	0.079	> 0.999	0.04
		For HIIT: Δ Late Tet – Δ Late Nontet	0.060	0.074	0.008*	0.81
		For HIIT: Δ Early Tet – Δ Late Tet	− 0.134	0.123	< 0.001**	1.09
AO	Central Occipital	For Rest: Δ Early – Δ Late	− 0.120	0.094	< 0.001**	0.96
		For HIIT: Δ Early Nontet – Δ Late Nontet	− 0.107	0.108	< 0.001**	0.99
		For HIIT: Δ Early Tet – Δ Early NonTet	− 0.009	0.079	> 0.999	0.11
		For HIIT: Δ Late Tet – Δ Late NonTet	0.063	0.078	0.008*	0.82
		For HIIT: Δ Early Tet – Δ Late Tet	− 0.179	0.133	< 0.001**	1.35

*ROI* region of interest, *SD* standard deviation, *d* Cohen’s d effect size, *AE* aperiodic exponent, *AO* aperiodic offset, Δ change relative to baseline, *Baseline* baseline time point, Early early post-tetanus time point, *Late* late post-tetanus time point, *Nontet* non-tetanised stimulus, Tet tetanised stimulus.

Data are collapsed across levels of unstated factors.

All p-values reported are Bonferroni corrected.

*p =  < 0.05.

**p =  < 0.001.

**Figure 3:**
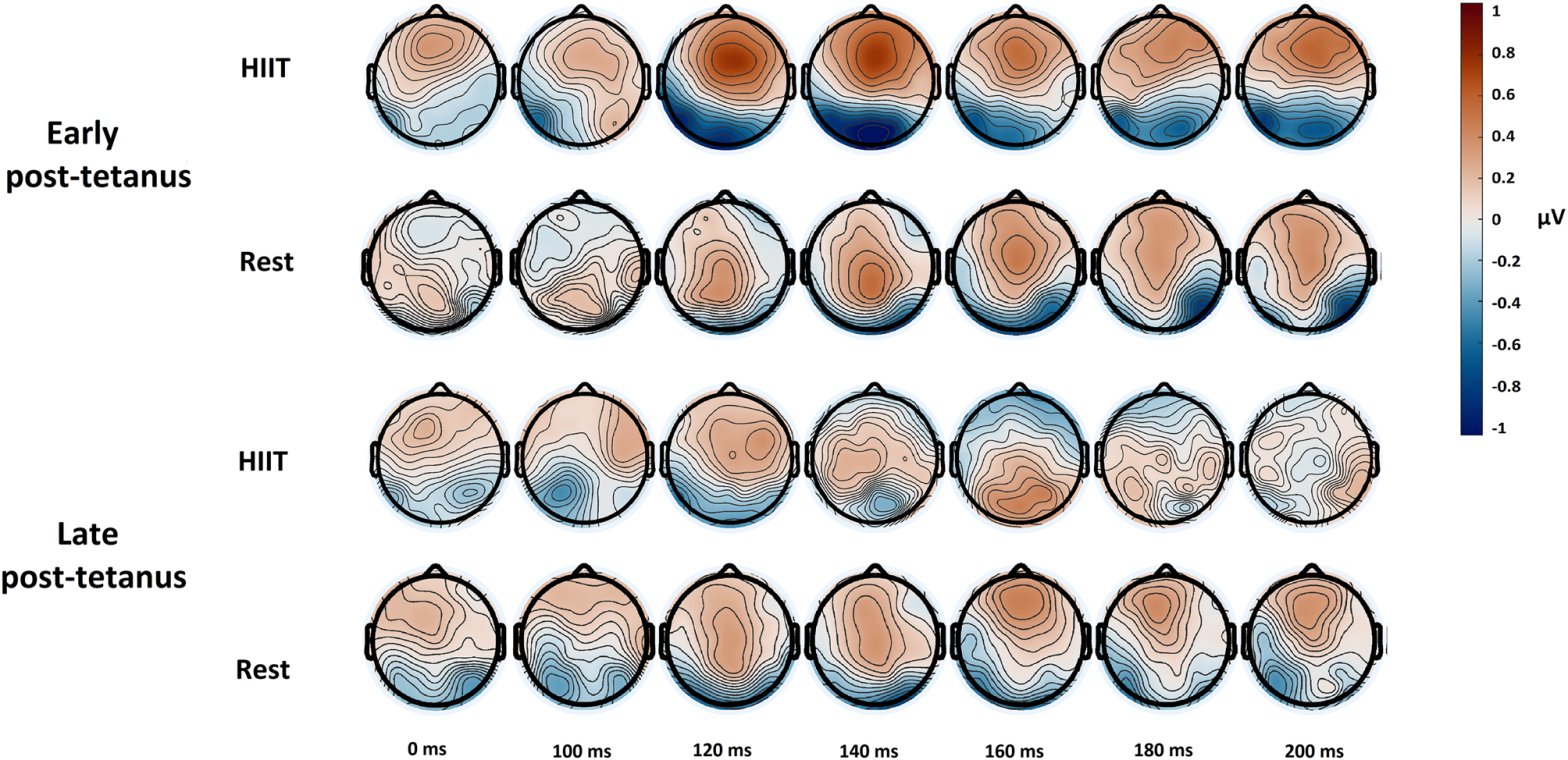
Scalp maps display the VEP amplitude change relative to baseline, for the tetanised stimulus minus the non-tetanised stimulus. Note that in the early post-tetanus time there appears to be a larger increase in negativity in the HIIT session compared to the rest session. Cluster-based permutation testing did not reveal significant effects.

Scalp maps representing the VEP data across time are shown in Fig. 3. Consistent with there being no significant effect of HIIT exercise for the VEP data in the ANOVA, the cluster-based permutation test was not statistically significant.

### P2

**Figure 4:**
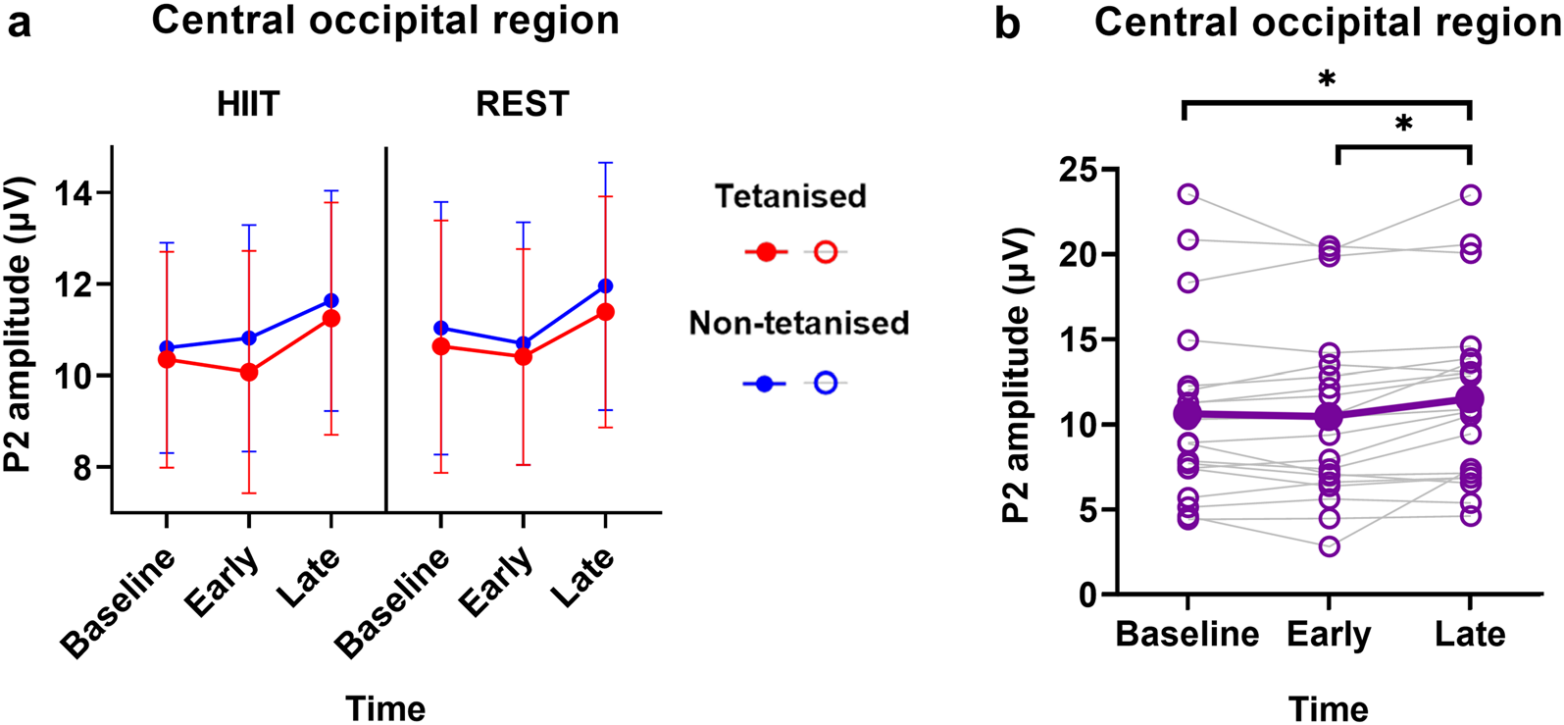
(**a**) P2 amplitude in the central occipital region. (**b**) P2 amplitude change across time. Individual data points are plotted. Data is collapsed across levels of Session and Stimulus. *p < .05; error bars show 95% confidence interval.

The ANOVA for the P2 amplitude from the central occipital region revealed a main effect of Time (baseline, early post-tetanus, late post-tetanus), F(2,38) = 7.250, p = 0.002, η^2^p = 0.276 (Fig. 4a). No other effects were significant (all p > 0.079). To explore this effect, the data were collapsed across the levels of Session and Stimulus, and three paired t-tests were conducted comparing all combinations of the levels of Time (Table 2). As expected, the P2 amplitude increased at the late post-tetanus time point, compared to both the baseline (p = 0.014) and early post-tetanus (p = 0.003) time points (Fig. 4b).

### Aperiodic exponent

The ANOVA for the aperiodic exponent in the left and right LO regions did not reveal any significant effects (all p > 0.125).

**Figure 5:**
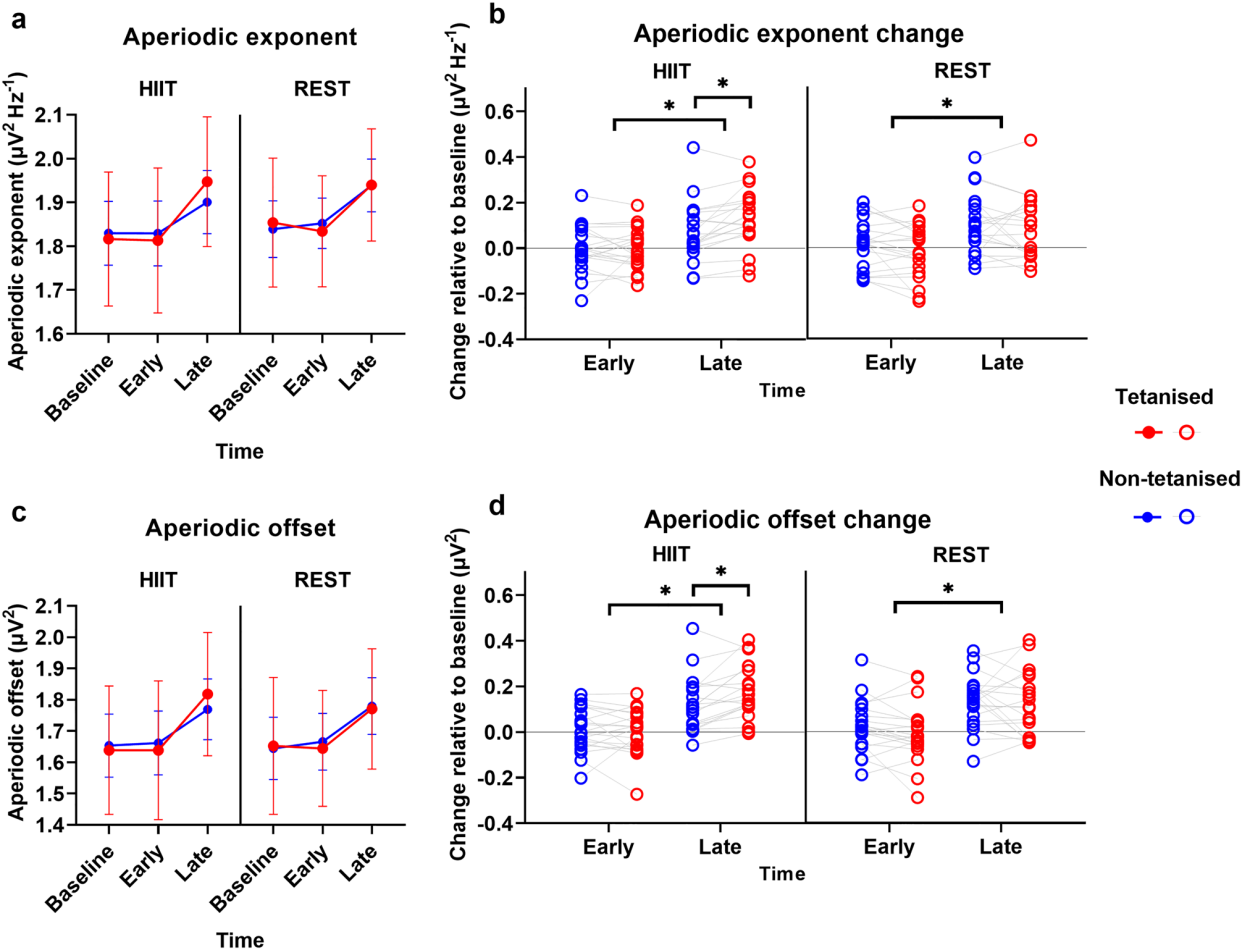
Aperiodic exponent and offset results in the central occipital region (electrode POz). (**a**) Aperiodic exponent. (**b**) Change in the aperiodic exponent relative to baseline. Individual data points are plotted. (**c**) Aperiodic offset. (**d**) Change in the aperiodic offset relative to baseline. Individual data points are plotted. *p < .05; error bars show 95% confidence interval.

The ANOVA for the central occipital region revealed a three-way interaction between Session (HIIT and rest), Stimulus (tetanised and non-tetanised) and Time (baseline, early post-tetanus and late post-tetanus), F(1.725, 32.769) = 4.665, p = 0.021, η^2^p = 0.197 (Fig. 5a). Prior to exploring this interaction, two paired t-tests were conducted as a manipulation check to demonstrate that there were no baseline differences between the HIIT and rest session for either stimulus (p > 0.504). Subsequently, the aperiodic exponent data at baseline was subtracted from the early post-tetanus and late post-tetanus time points. The magnitude of change (relative to baseline) for the tetanised and non-tetanised stimulus was compared by conducting two 2 × 2 ANOVAs with Stimulus (tetanised and non-tetanised) and Time (early post-tetanus and late post-tetanus), for each Session (HIIT and rest) separately.

The ANOVA for the rest session revealed a significant main effect of Time, F(1, 19) = 19.984, p =  < 0.001, η^2^p = 0.513. There was no effect of Stimulus. The post-hoc t-test (Table 2) revealed that the increase in the aperiodic exponent (relative to baseline) was greater at the late post-tetanus time point, compared to the change seen at the early post-tetanus time point (p < 0.001; Fig. 5b). The ANOVA for the HIIT session revealed a main effect of Time, F(1,19) = 17.870, p =  < 0.001, η^2^p = 0.485, and a two-way interaction between Stimulus and Time, F(1,19) = 12.307, p = 0.002, η^2^p = 0.393. Post-hoc t-tests (Table 2) revealed that, consistent with the rest session, the increase in the aperiodic exponent (relative to baseline) was greater at the late post-tetanus time point, compared to the early post-tetanus time point for both the tetanised (p < 0.001) and non-tetanised stimulus (p = 0.032). Importantly, the magnitude of this late post-tetanus increase was greater for the tetanised stimulus, compared to the non-tetanised stimulus (p = 0.008; Fig. 5b).

### Aperiodic offset

The ANOVA for the aperiodic offset in the left and right LO regions did not reveal any significant effects (all p > 0.056).

The ANOVA for the aperiodic offset in the central occipital region revealed a three-way interaction between Session (HIIT and rest), Stimulus (tetanised and non-tetanised) and Time (baseline, early post-tetanus and late post-tetanus), F(1.547, 29.395) = 4.917, p = 0.021, η^2^p = 0.206 (Fig. 5c). Prior to exploring this interaction, two paired t-tests were conducted as a manipulation check to demonstrate that there were no baseline differences between the HIIT and rest session for either stimulus (p > 0.803). Subsequently, the aperiodic offset data at baseline was subtracted from the early post-tetanus and late post-tetanus time points. Two 2 × 2 ANOVAs were conducted with Stimulus (tetanised and non-tetanised) and Time (early post-tetanus and late post-tetanus), for each Session (HIIT and rest) separately.

The results of these ANOVAs showed the same pattern of results seen for the aperiodic exponent. The ANOVA for the rest session revealed a main effect of Time, F(1,19) = 31.682, p =  < 0.001, η^2^p = 0.625. The post-hoc t-test (Table 2) revealed that the increase in the offset (relative to baseline) was greater at the late post-tetanus time point, compared to the change seen at the early post-tetanus time point (p < 0.001; Fig. 5d). The ANOVA for the HIIT session revealed a main effect of Time, F(1,19) = 32.174, p =  < 0.001, η^2^p = 0.629, and a two-way interaction between Stimulus and Time, F (1,19) = 13.570, p = 0.002, η^2^p = 0.417. Post-hoc t-tests (Table 2) revealed the increase in offset (relative to baseline) was greater at the late post-tetanus time point, compared to the early post-tetanus time point for both the tetanised (p < 0.001) and non-tetanised stimulus (p < 0.001). The magnitude of this late post-tetanus increase was greater for the tetanised stimulus, compared to the non-tetanised stimulus (p = 0.008; Fig. 5d).

## Discussion

In this study we tested the hypothesis that an acute bout of HIIT exercise would augment LTP in the visual cortex. We replicated the LTP-like potentiation of the N1b and P2 following a visual tetanus^17–20^, however, contrary to our hypothesis, there was no effect of a preceding bout of HIIT exercise on these visual LTP markers. We further investigated changes in the EEG-derived aperiodic exponent, an index of the cortical E:I balance. In the central occipital region, there was an increase in the aperiodic exponent in the late post-tetanus time period, suggesting a late shift in the E:I balance towards inhibition. While there was no stimulus specificity seen for this effect following a preceding bout of rest, HIIT exercise resulted in specificity of this inhibitory shift in the E:I balance to the tetanised stimulus compared to the non-tetanised stimulus. This suggests that HIIT exercise enhanced specificity of the post-tetanus E:I balance shift in the visual cortex to stimulus details present during LTP induction.

### Acute exercise and markers of visual LTP

In contrast to previous TMS studies demonstrating an increased magnitude of LTP following acute HIIT exercise in a non-exercised area of the motor cortex^11,13^, the current study found no effect of HIIT exercise on markers of visual LTP. It is possible that acute exercise has region-specific effects, and that we see significant findings in the motor cortex due to its proximity to areas of the brain that were very active during exercise. Alternatively, the lack of effect in this study may be attributed to the delay (~ 40 min) between exercise and LTP induction due to the EEG cap preparation. In those studies that have previously demonstrated an exercise-related LTP augmentation, LTP induction occurs approximately 10–20 min after exercise. The acute neurochemical changes that are likely to create a favourable environment for LTP following exercise peak immediately after exercise, and progressively decline over time^34^. Therefore, LTP induction in the current study may have occurred after the optimal time window for maximal acute exercise effects. While we are the first to explore the impact of acute exercise on visual LTP in humans, others have shown that adults who engage in high levels of regular exercise show an increased magnitude of LTP in the visual cortex compared to sedentary adults^35^. This supports the idea that regular exercise and physical fitness have effects on visual LTP, possibly via the accumulative effects of repeated bouts of acute exercise.

### Post-tetanus changes in the E:I balance

To our knowledge, this study is the first to report changes in aperiodic activity following LTP induction by visual tetanus in humans. Here we show a late post-tetanus increase in the aperiodic exponent, indicating a shift in the E:I balance towards inhibition following a visual tetanus. This shift showed a similar topography and latency to the LTP-like potentiation of the P2 in this study. These results are in contrast to previous findings in the motor cortex of an immediate shift in the E:I balance towards excitation following LTP induction with TMS^13^. Furthermore, this result contrasts with studies investigating the effects of pharmacological intervention on visual LTP. Drugs hypothesised to increase cortical excitability, such as 3-h post ketamine infusion and acutely following a dose of d-cycloserine, have a facilitatory effect on visual LTP^36,37^. However, there is evidence that increases in inhibitory activity occur in tandem with excitatory changes at different cellular layers of the visual cortex following LTP induction with a visual tetanus^38^. It is possible that previous contrasting findings are a result of different mechanisms being captured by different methods of understanding excitatory and inhibitory activity (e.g. TMS, the aperiodic exponent and drug manipulations). The observed inhibitory shift in E:I balance in the current study was accompanied by an increase in broadband power, indexed by an increase in the aperiodic offset. Previous studies have linked increases in broadband power to increased neuronal population spiking^24^. The inhibitory shift in the E:I balance observed in the visual cortex in our study may therefore, be driven primarily by an increase in inhibitory activity, rather than a decrease in excitatory activity. For stronger conclusions to be drawn, further research exploring the links between the neural mechanisms captured by these aperiodic measures is required.

### An acute exercise-related modulation of the E:I balance

Importantly, we showed that a preceding bout of acute HIIT exercise resulted in stimulus specificity of the inhibitory E:I balance shift following the visual tetanus, which was not shown in the rest condition. This exercise-related specificity to the tetanised stimulus showed a large effect size (Cohen’s d = 0.81). We propose that this may represent a tuning of the cortical response to visual stimulus details following LTP induction. Tuning of the visual cortex is a process whereby the precision of neural representations is enhanced. Adjustment of the E:I balance is achieved by a fine modulation of inhibitory populations (e.g. parvalbumin-positive interneurons) to facilitate a selective suppression of undesired neural activity for signal contrast enhancement^39,40^. It has been demonstrated in the rodent and feline brain^39–41^ and shown to enhance perceptual discrimination^40^.

Although previous studies in the motor cortex of humans have shown that acute HIIT exercise is associated with an increase in excitatory mechanisms in the motor cortex using TMS measures^11,13,16^, Maddock and colleagues^7^ report a long-lasting increase in the concentration of the inhibitory neurotransmitter gamma-aminobutyric acid (GABA) in the visual cortex following an acute bout of HIIT exercise as measured with magnetic resonance spectroscopy (see also Ref.^30^). This suggests that while exercise may induce excitatory changes in at the synaptic level, as measured by TMS^11,13^, the exercise-related enhancement of the inhibitory E:I balance shift shown in the current study may be more reflective of the overall increase in GABA concentration in the visual cortex following exercise^42^. While the reported exercise effect is a promising finding, some caution is required when interpreting it in the context of LTP due to the absence of change in overall LTP magnitude following exercise in this study.

### Strengths, limitations and future directions

The current study did not include pre-exercise measurements to allow a direct pre- to post-exercise comparison before LTP induction. Instead, where significant effects of exercise were observed, the comparison of baseline data between the HIIT and rest session for each participant was made. As expected, these did not yield significant differences, supporting the assumption that exercise in isolation does not cause direct changes in the electrophysiological measures of interest^13^. Future areas of investigation include the possible impact of factors known to influence LTP and the E:I balance, such as the BDNF Val66Met polymorphism^13,43^, and cardiorespiratory fitness^35^, on the magnitude of changes in LTP and the E:I balance in the visual cortex following an acute bout of exercise.

Finally, this study highlights the methodological strength of measuring aperiodic activity changes when investigating LTP with the visual LTP paradigm. Previous studies have failed to demonstrate stimulus specificity of the P2 potentiation following a visual tetanus, a key feature of LTP^20,22^. In this study we showed that there are coinciding changes in aperiodic measures that share a similar latency and topography to the P2, and that demonstrate some stimulus specificity. This suggests that aperiodic measures may be capturing additional information about LTP. Future studies using this paradigm should consider using the FOOOF toolbox to gain further information about changes in LTP-related mechanisms.

## Conclusion

Our hypothesis of an enhancement of visual LTP following an acute bout of exercise was not supported as there was no effect of HIIT exercise on N1b or P2 potentiation. However, here we report the first evidence that exercise modulates aperiodic EEG activity, with specificity of the E:I balance shift in the visual cortex to stimulus characteristics present during LTP induction. We interpret this as an exercise-induced effect on visual tuning, an effect that was not seen following rest. The functional importance of this exercise-facilitated tuning has possible implications for visual recognition of stimulus details.

## Data Availability

The datasets analysed during the current study are available in the Figshare repository, https://doi.org/10.26180/21737966.v1.
